# On the availability of microRNA-induced silencing complexes, saturation of microRNA-binding sites and stoichiometry

**DOI:** 10.1093/nar/gkv720

**Published:** 2015-07-30

**Authors:** Vinay K. Mayya, Thomas F. Duchaine

**Affiliations:** Department of Biochemistry & Goodman Cancer Research Centre, McGill University, Montreal, Quebec H3A 1A3, Canada

## Abstract

Several authors have suggested or inferred that modest changes in microRNA expression can potentiate or impinge on their capacity to mediate gene repression, and that doing so could play a significant role in diseases. Such interpretations are based on several assumptions, namely: (i) changes in microRNA expression correlate with changes in the availability of mature, functional miRISC, (ii) changes in microRNA expression can significantly alter the stoichiometry of miRISC populations with their cognate targets, (iii) and this, in turn, can result in changes in miRISC silencing output. Here, we experimentally challenge those assumptions by quantifying and altering the availability of miRISC across several families of microRNAs. Doing so revealed a surprising fragmentation in the miRISC functional pool, striking differences in the availability of miRNA families and saturability of miRNA-mediated silencing. Furthermore, we provide direct experimental evidence that only a limited subset of miRNAs, defined by a conjuncture of expression threshold, miRISC availability and low target site abundance, is susceptible to competitive effects through microRNA-binding sites.

## INTRODUCTION

MicroRNAs (miRNAs) are ∼22 nucleotide (nt)-long RNAs, which regulate a broad variety of biological processes by impinging on gene expression. Thus far, close to 500 miRNA genes have been identified in the human genome (1) and they are suspected to regulate more than 60% of protein-coding genes (2). When embedded into Argonaute proteins (AGO1–4 in mammals) as part of the miRNA Induced Silencing Complex (miRISC), miRNAs direct target recognition through partial base-pairing with sites most often located in 3′-untranslated regions (3′ UTRs). This initiates a series of events that culminate with the translational repression and the destabilization of target mRNAs (3–5). The underlying mechanism involves deadenylation, de-capping and other activities that are scaffolded onto miRISC via the GW182 proteins (TNRC6 in mammals).

The extent of mRNA silencing mediated by miRNAs varies greatly and the reasons for such a disparity are not fully understood. Silencing is sensitive to several constraints and parameters, including the sequence, structure, density and distribution of miRNA-binding sites in an mRNA (6). Evidence is mounting that miRNA-mediated silencing can be further modulated through several context-dependent mechanisms. One such mechanism with far-reaching implications was postulated by the competing endogenous RNA (ceRNA) hypothesis (7,8), whereby co-expressed RNA species, including mRNAs and long non-coding RNAs such as pseudogenes or circular RNAs (9,10), affect target mRNA silencing by competing for a common pool of miRNAs. A central prediction of this hypothesis is that changes in the availability of miRNAs, in contrast with their expression alone, could alter the potency of target mRNA silencing. Prior experimental evidence indeed appeared to support this possibility: ectopic expression of RNAs encoding multiple binding sites for a particular miRNA (often called miRNA sponges) could de-repress endogenous and reporter miRNA targets (11,12). Since then, several studies have interpreted both correlative and anti-correlative changes in expression of miRNAs and their target mRNAs in light of the ceRNA hypothesis. In some cases, coordinated changes in miRNA, ceRNA and mRNA expression were suspected to play a critical role in diseases including cancer (13–15).

Several recent initiatives have turned to directly test the ceRNA hypothesis, both theoretically and experimentally, and identified some of its limitations. An emerging conclusion is that specific conditions of abundance and stoichiometry must be met for changes in competing RNA expression to affect miRNA-mediated silencing. For example, competition for miRNAs is predicted to be maximal when the concentration of targets and miRNA is nearly equal (16–18). Conversely, target competition effects can fail due to high abundance of miRNA-binding sites (19). Such interpretations are in line with a genome-wide assessment of the output of miRNAs, which revealed that only a fraction of the most abundant miRNAs, a select group characterized by low predicted target site-to-miRNA ratio, exert significant silencing (20). Most recently, an elegant integration of gene expression, Argonaute iCLIP datasets and modelized target site affinities further indicated that only those miRNA families expressed at low target site-to-miRNA ratio are susceptible to target site competition effects (21). Notwithstanding such insightful studies, decisive determination of effective stoichiometry of miRNAs and target sites remains a challenge. On one hand the cumulative concentration of target sites is modelized or inferred, and on the other hand effective miRISC concentration is affected by biochemical and sub-cellular compartmentalization.

We reasoned that direct empirical measurement of miRISC availability could better substantiate and refine the emerging views on critical stoichiometric aspects of miRNA-mediated silencing. Here, we sought to directly assess the relationships between miRISC availability, miRNA expression and silencing outcome across a diverse set of cancer-linked miRNA families. Using quantitative target analogue-based miRISC capture, absolute quantification of miRNAs and an array of reporter silencing assays, we demonstrate that miRISC availability is linked to, but distinct from miRNA expression, and greatly varies across miRNA families. Considering the availability of miRISC and its effects on silencing further refines the stoichiometric requirements for functional competition between co-expressed target RNAs, and unveils some of its key mechanistic bases.

## MATERIALS AND METHODS

### Plasmids

For the silencing assays, oligonucleotides encoding binding sites (1x-Perfect/ 3x-Bulge) [see Supplementary Table S1] for each of the miRNAs (IDT) were annealed and inserted downstream of Firefly luciferase gene between XBaI/NotI sites of the pmiRGLO vector (Promega). The construct used for ectopic expression of miR-19a/b was described and validated in (22). The Sponge-19 plasmid was a generous gift from Dr Kai Fu (University of Nebraska) (23).

### 2′-O-Me capture and western blot

HEK 293T cells cultured in Dulbecco's modified Eagle's medium supplemented with 10% foetal bovine serum (Wisent) and gentamycin were plated on 10-cm plates (BD Falcon). After 3 days, cells were collected and resuspended in ice-cold lysis buffer (25 mM HEPES-KOH pH 7.4, 120 mM NaCl, 1 mM ethylenediaminetetraacetic acid (EDTA), 2.5% glycerol, 0.5% Triton-X, 2 mM Dithiothreitol (DTT)), supplemented with protease and phosphatase inhibitor cocktail (Sigma). MyOne T1 streptavidin beads (25 μl; Invitrogen) were washed three times with 1× binding and washing buffer (10 mM Tris–HCl pH 7.5, 1 mM EDTA, 2 M NaCl). The slurry was then resuspended in the same buffer to obtain a final concentration of 5 mg/ml and 10 μl of specific 2′-O-Me oligonucleotide (10 μM) was added. After incubation at RT for 30 min., the beads were washed, 1.75 mg of protein lysate was added and the mixture was incubated in a rotating wheel for 15 min. The beads were again washed and resuspended in 2× sodium dodecyl sulphate buffer. To monitor the efficiency of RISC capture following incubation of specific 2′-O-Me oligonucleotides with the beads the protein lysate was added and the mixture was incubated in a rotating wheel for 120 min. The unbound fraction was then recovered and RNA was extracted using QIAzol (Qiagen) for northern analyses. For experiments involving expression of sponge and ectopic expression of miR-19a/b, the plasmids were transfected at 70–80% confluency for HEK 293T cells plated in 10-cm dishes. The expression of the sponge was induced the following day by addition of doxycycline (1 μg/ml) and cells were harvested on the next day (24 h).

For the detection of Argonautes, AGO1 (1:1,000) from cell signalling; AGO2 (1:1,000) from Abcam#135025; AGO1–4 (1:500) from Millipore #2A8 each diluted in blocking buffer were used (Odyssey, LiCor). Bound primary antibodies were detected using Goat anti-Rabbit IR dye (1:10,000) or Goat anti-Mouse IR dye (1:10,000) using an Odyssey imaging system from LiCor.

### Luciferase assay

At 70–80% confluency, HEK 293T cells plated on 24 wells were co-transfected with 10 ng of each of the pmiRGLO reporters and varying concentrations of miRNA inhibitors/mimics (Qiagen). After 48 h of transfection, cells were lysed and Firefly and *Renilla* luciferase activities were determined using dual luciferase kit (Promega, BioTek).

### Data and statistical analyses

IC_50_ values were obtained by normalizing F-Luc/R-Luc values of the WT reporter over the non-responsive (MUT) version. The curve was fit using four-parameter analysis with a Hill's constant = 1. All data are presented as mean ± standard deviation. Student's *t*-test was employed for comparisons between samples with *P < 0.05, **P < 0.005.

### Quantitative northern blot analysis

Standard RNA oligos of miR-19b, miR-20a, miR-26a, miR-92a, let-7a, let-7b, miR-17, miR-20b, miR-25, miR-93 and miR-106b obtained from Integrated DNA Technologies (IDT) were each diluted to concentrations of 1, 5, 10, 15, 20, 25, 30 and 40 pg/μl. Total RNA (2.75/5.5 μg) from HEK 293T cells and the standards were resolved on 15% TBE-Urea gel (Bio-Rad), transferred onto to Hybond-XL membrane (Amersham) and UV-crosslinked. Hybridization was carried out using ^32^P-labelled Starfire probes (miRfire IDT) at 25°C overnight in ULTRAHyb Oligo Hybridization buffer (Ambion). Following hybridization, membranes were washed, exposed onto an imaging plate (Fujifilm) and developed using a phosphoimager (Typhoon). Intensity of the signal was quantified using ImageJ software.

### qRT-PCR analysis

Synthetic RNA oligonucleotides were used as quantitative standards and the absolute levels of miRNAs were quantified as described in (24). Templates for *in vitro* transcription of F-Luc and Sponge-19 RNAs were prepared by polymerase chain reaction (PCR) using primers encoding T7 RNA polymerase promoter sequence [see Supplementary Table S1]. Following *in vitro* transcription (Ambion), the samples were treated with DNase I and purified using RNA spin column (Roche). Reverse transcription of known amounts of *in vitro* transcripts RNA spiked in HEK 293T RNA was performed (Bio-Rad) alongside RNA extracted from cells expressing 3x-Bulge-miR-19 and the Mutated (MUT) version of the reporter. qPCR was performed using a SYBR green-based method (Qiagen) [see Supplementary Table S1 for primer pairs]. Quantitation of F-Luc and Sponge-19 mRNAs was then determined using the standard curve obtained using *in vitro* transcribed templates.

## RESULTS

**Figure 1.**
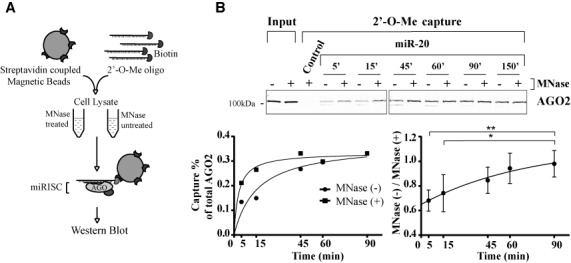
Quantitative capture of available miRISC using target site analogue oligonucleotides (2′-O-Me pull-down). (**A**) Schematic representation of the capture strategy. Biotinylated 2′-O-methylated (2′-O-Me) oligonucleotides encoding a binding site for a particular miRNA family (here, miR-20 and a *Caenorhabditis elegans* specific miRNA, miR-35 as a control) are bound to streptavidin-coupled magnetic beads and incubated for a defined time in HEK 293T protein lysates pre-treated or un-treated with MNase. Beads are pulled out, washed and western blot is performed against AGO2, a core component of miRISC. (**B**) Bottom left panel: quantification of the presented AGO2 western blot. Bottom right panel: aggregate quantification from four independent biological replicate experiments. Data is presented as a ratio of untreated (MNase (−)) over MNase-treated (MNase (+)). Quantitative imaging was performed using the Odyssey imaging system (LiCor). A two-tailed Student's *t*-test was used to calculate *P*-values (*: *P* < 0.05, **: *P* < 0.005).

To profile the available population of miRISC, we used a strategy based on streptavidin-affinity capture of biotinylated 2′-O-methyl oligonucleotides (2′-O-Me), which mimic miRNA target sequences and efficiently capture paralogous miRNAs (with the same seed sequence) (25–27) (Figure 1A). First, miRISC capture was conducted for the miR-20 family in HEK 293T lysate over a 150-min time-course. As a surrogate of miRISC, we detected Argonaute AGO2 (a core protein in miRISC) by western blot. For all capture experiments, similar results were observed when blotting for AGO1 or using a pan-AGO antibody that detects all the four human Argonautes (28) [Supplementary Figure S1A, B]. miRISC capture was progressive over the time course and reached a plateau between 45 and 60 min of incubation (Figure 1B). At this time, the miR-20 2′-O-Me target analogue captured 0.3% of total AGO2 in the lysate (Figure 1B, bottom left panel). We reasoned that this progressive binding might be reflective of a mixture of miRISC populations: a fraction already associated to cognate and/or non-cognate mRNA molecules and a second available fraction, readily capturable. To test this hypothesis, we pre-treated the lysate with MNase, which preserves miRNAs but not mRNA targets (29) and proceeded with the same capture time course. Quantification of four independent biological replicates indicates that both capture conditions yielded the same maxima at late time points, but capture is significantly accelerated by treatment with MNase (Figure 1B, bottom right panel). These results suggest that target analogue capture is sensitive to the availability of miRISC in cell lysates, with free miRISC being captured faster than target RNA-bound miRISC. As the 15-min time point was the most reflective of the available pool of miRISC, later captures were carried out at this time point.

**Figure 2.**
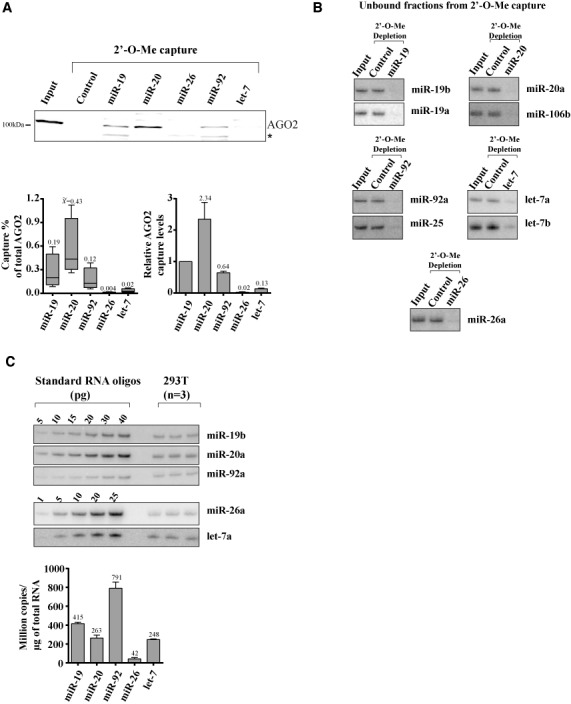
Comparative quantitation of miRNA expression and miRISC capture for the miRNA families considered in this study. (**A**) miRISC capture was conducted as above, for the miR-19, -20, -92, -26 and let-7 families. Captured AGO2 was quantified from four independent biological replicates (bottom panels). Results are presented as percentage of total AGO2 in HEK 293T lysate input (10 μg) (box plot, bottom left, median values [x with tilde] indicated above) and normalized against miR-19 capture for each individual lysate (bottom right, average values indicated above (**B**) northern blot analyses of unbound miRNA fractions recovered after miRISC capture. (**C**) Quantitative northern blot analyses of each miRNA for three independent biological replicates. RNA oligonucleotides were used as standards and copy numbers were calculated per microgram of isolated RNA (bottom, average values indicated above). All other paralogous miRNAs examined were less abundant (Supplemental Materials). The asterisk (*) indicates a non-specific band.

miRNA expression profiles reflect a wide variety of intracellular and environmental cues, which are unique to cellular and organism contexts. We asked how the readily capturable miRISC pool relates to miRNA expression levels. miRISC capture was compared for miR-19, miR-20, miR-92 families, members of which are encoded in the proto-oncogenic miR-17∼92 polycistron, as well as let-7 and miR-26 miRNAs (Figure 2A). Major differences in capture were noticeable between miRNAs: a higher percentage of miRISC was captured using the miR-20 target analogue (median 0.43% of total AGO2 in lysates (*n* = 4)) than miR-19 (0.19%) and miR-92 (0.12%) families (Figure 2A, box plot, bottom left panel). miR-26 did not capture miRISC significantly above background, whereas let-7 captured 0.02% of total AGO2 under the same conditions. While some spread of values is visible in box plots for individual captures of each miRNA family across independent lysates, the relative AGO2 capture of miRNA families remained strictly consistent in each individual lysate (lower right panel). Differences between miRNA families were unlikely due to selective small RNA sorting in distinct Argonautes, as evidence strongly argues against such a process in mammalian cells (30,31). 2′-O-Me baits were utilized in excess and when capture was carried out for 2 h, comparable depletion was achieved across- and within each miRNA families (Figure 2B and Supplementary Figure S2A and B). Capture of miR-19, miR-20, miR-92, miR-26 and let-7 paralogous miRNAs was largely independent of mismatches with central and 3′ miRNA sequences and was primarily dictated by seed complementary sequence.

We next set out to compare miRISC capture with miRNA abundance. Next-generation sequencing has proven invaluable for the identification and relative quantitation of miRNAs in different conditions, but its use in absolute quantitation without internal standards is limited by biases in library preparation (32–34). Instead we opted for standardized northern blots to quantify the expression of miRNAs. These analyses suggest that miR-92a is the most abundant of the examined miRNAs in HEK 293T, at 791 × 10^6^ copies/μg total RNA, ahead of miR-20a (263 × 10^6^ copies/μg total RNA), miR-19b (415 × 10^6^ copies/μg total RNA) and let-7a (248 × 10^6^ copies/μg total RNA) (Figure 2C, Supplementary Figure S3A). In comparison, their paralogues miR-17, miR-20b, miR-25, miR-93, miR-106b, let-7b, often abundantly detected in sequenced libraries (35,36), were expressed at very low copy numbers or were undetectable (Supplementary Figure S3B). Paralogue-specific detection using this method has been validated previously (25) and was further confirmed using total RNA isolated from mir-17–92 deletion MEFs (Supplementary Figure S3C). Quantitative northern results were further verified using standardized, paralogue-specific, LNA-based qRT-PCR assays on miRNAs (24) Supplementary Figure S3 D and E). Strikingly, although miR-19, miR-20 and let-7 miRNAs are expressed at comparable levels, they exhibited marked differences in miRISC capture assays. miR-20 is approximately three-fold less abundant than miR-92, its expression being comparable to miR-19, whereas its capture yields 2.3-fold more miRISC than miR-19 and 3.7-fold more than miR-92. Furthermore, whereas let-7a and miR-20a are expressed at comparable levels in HEK 293T cells, capture of miR-20 yields 117-fold more miRISC than let-7. Altogether, these results point to divergences between the expression levels of miRNAs and their functional availability as part of miRISC.

**Figure 3.**
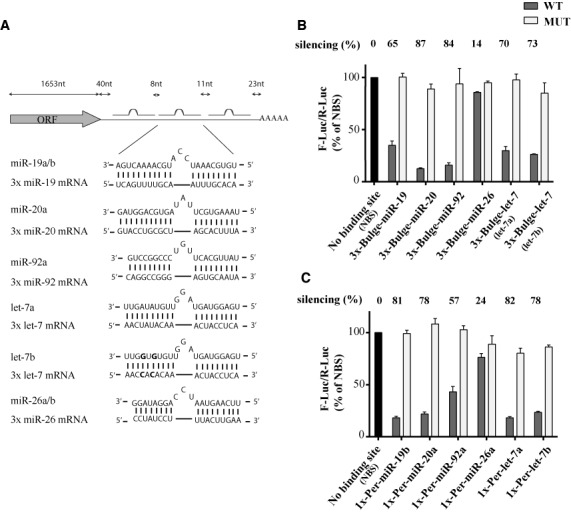
Comparative slicing and slicing-independent silencing by miRNA families. (**A**) Schematic representation of 3x-Bulge Firefly luciferase (F-Luc) silencing reporters. The structures of mismatched (Bulge) sites are described. Spacing and positioning of sites is the same for all reporters [though miR-26 reporter has a minor disparity] (See Supplementary Table S1). Mutated (MUT) reporters encode an additional 3-nt mutation in seed-complementary sequences (See ‘Materials and Methods’ section). Differences between the let-7a and let-7b-based reporters are highlighted in bold. (**B**) HEK 293T cells were co-transfected with constructs expressing WT or MUT sites (as indicated) for miR-19, miR-92, miR-20, miR-26 or let-7, and *Renilla* luciferase (R-Luc) as an internal standard. Normalized counts are compared to the no binding site reporter (NBS), which is set as 100% for comparison. (**C**) Similar analyses were conducted with reporters expressing one perfectly matching site for each examined miRNA. All data are presented as mean ± standard deviation from technical triplicates of three independent biological experiments.

Having profiled the expression and availability of the miR-19, 20, 92, 26 and let-7 miRNAs, we next compared their silencing output. As miRNA target sequences intricately interact with RNA structures and RNA binding proteins within endogenous 3′ UTRs, we opted for simple, well-controlled reporters to specifically contrast the impact of miRNAs. Firefly luciferase (FLuc) reporters were built to encode, in their 3′ UTR, precisely positioned binding sites for each of the miRNAs. One version encoded three site copies with pairing mismatches at positions 9, 10 and 11, to prevent endo-nucleolytic (slicing) activity by AGO2, thus enabling the monitoring of miRNA-mediated repression (37) (Figure 3A and B). A second version encoded a single, fully base-pairing site to monitor the slicing activity of miRISC (Figure 3C). Constructs were transfected in HEK 293T and the potency of their silencing was determined and compared using *Renilla* luciferase (RLuc) activity as an internal reference. A let-7b-based reporter led to silencing virtually indistinguishable from a let-7a-based design, in spite of mismatches in sequence complementary to the paralogues 3′ end. Furthermore, reporters bearing mismatches in the seed complementary region (bases 3–5) led to counts indistinguishable from the *No binding site* (NBS) reporter (Figure 3B, C; MUT and NBS lanes). These results indicate that our reporters account for silencing by families of paralogous miRNAs and reflect specificity as defined by the seed sequence. The extent of miRNA-mediated silencing significantly varied between miRNAs. While it is less abundantly expressed, the more available miR-20 miRNAs silenced cognate 3x-Bulge reporter by 87%, significantly outperforming miR-19 (65%) and miR-92 (84%) (Figure 3B). let-7 silenced its cognate reporter to an extent comparable with miR-19 (70 versus 65%), in spite of its limited availability.

Single, perfect site reporters (1xPer lanes, Figure 3C) also led to potent silencing, but with virtually the same silencing by miR-19, 20 and let-7 miRNAs (78–82%) in spite of the differences in their expression and availability. In contrast with the potent silencing exerted upon the 3x-Bulge-miR-92 reporter, its 1xPer counterpart was silenced less (57%) than for the other detected miRNAs. However, for both 3x-Bulge-miR-26 and 1xPer reporter, silencing was negligible compared to the other miRNAs. Altogether, these results indicate that neither the miRNA expression level, nor their availability alone, can predict the extent of reporter silencing through slicing or non-slicing mechanisms.

**Figure 4.**
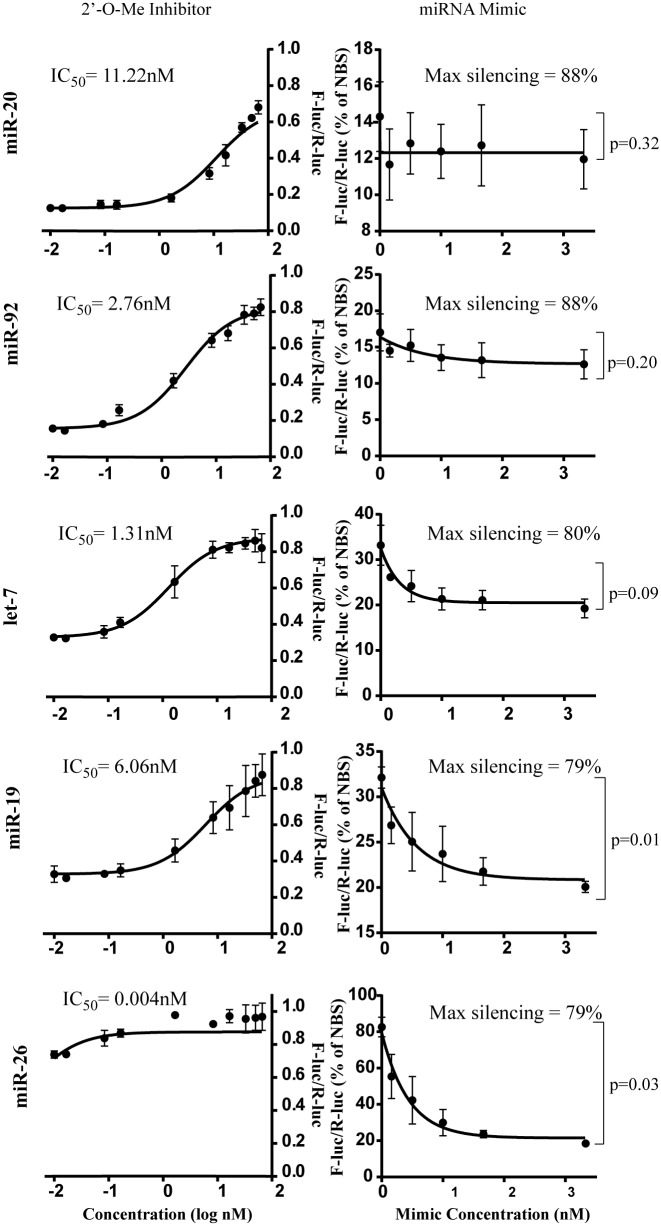
Quantitative assessment of the effect of miRISC availability on miRNA reporter silencing. (Left panel) 3x-Bulge reporters were co-transfected with increasing concentrations (0–66nM) of 2′-O-Me inhibitors for the indicated miRNAs. The concentrations of inhibitors required to reach half-maximal inhibition (IC_50_) for each reporter was calculated by normalizing FLuc-3x-Bulge (WT)/R-Luc ratios to FLuc-3x-Bulge (MUT)/R-Luc ratios and fitting the values into a sigmoidal curve, using four-parameter analyses. (Right panel) 3x-Bulge reporters were co-transfected with increasing concentrations (0–3.3 nM) of dsRNA miRNA mimics for the indicated miRNAs. FLuc-3x-Bulge/RLuc ratios are compared to No binding site reporter (NBS)/RLuc, set as 100%. All data are presented as mean ± standard deviation from technical triplicates of at least three independent biological experiments. Two-tailed Student's *t*-test was used to calculate *P*-values.

To better discern the interplay of stoichiometric parameters in miRNA-mediated silencing, we elected to experimentally alter the availability of miRISC for each miRNA family along a continuum of controlled concentrations while following the impact on reporter silencing. For this, 3x-Bulge reporters were co-transfected with increasing concentrations of 2′-O-Me inhibitors to titrate miRISC availability (Figure 4, left panel, inhibitors) and dsRNAs that mimic endogenous miRNAs to increase it (Figure 4, right panel, miRNA mimics). Strikingly, miRNA families exerted qualitatively and quantitatively distinctive response profiles to miRNA inhibitors and mimics. miR-20 and miR-92 reporters were progressively de-repressed with increasing concentrations of cognate miRNA inhibitors, but addition of increasing concentrations of mimics did not lead to significantly more silencing, indicating saturation. miR-19 and let-7 reporters were responsive to both de-repression by miRNA inhibitors and enforced reporter repression by miRNA mimics. At the opposite end of the spectrum from miR-20, and consistent with its very low abundance, the miR-26 reporter was not significantly de-repressed by the addition of inhibitor, but its silencing was strongly improved (by more than 60%) with the miR-26 mimic, reaching saturation at 2–3 nM. Importantly, neither inhibitors nor mimics affected MUT or NBS reporter expression within the range of concentrations examined (Supplementary Figure S4). Interestingly, reporters reached saturation under high endogenous and/or mimic miRISC concentrations, but reached different maxima. miR-20 and miR-92 reached maxima of silencing at 88 and 86% respectively, more than let-7 (78%), miR-19 (79%) and miR-26 (80%). Finally, IC_50_ values for 2′-O-Me inhibitors were significantly different between the miRNA families and followed the relative order of miRISC availability reflected in capture experiments (Figure 2A): miR-17/20 (IC_50_ 11.22 ± 0.61 nM) > miR-19 (IC_50_ 6.06 ± 1.17 nM) > miR-92 (IC_50_ 2.76 ± 0.21 nM) > let-7 (IC_50_ 1.31 ± 0.63 nM) >>> miR-26. These results again confirm the disparity between miRNA expression and the availability of miRISC and further suggest the functional fragmentation of miRNA/miRISC pools (see ‘Discussion’ section).

**Figure 5.**
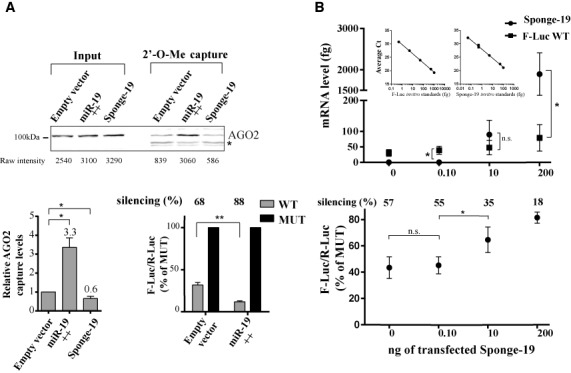
Near-stoichiometric competing effects for miRNA targets. (**A**) miRISC captures and reporter silencing were quantified for lysates derived from cells co-expressing ectopic miR-19a/b (miR-19++) or a transcript encoding miR-19-binding sites (Sponge-19), with the 3x-Bulge-miR-19 reporter. Captured AGO2 signal was normalized to input and compared with capture from a lysate co-expressing FLuc without UTR (‘Empty’, bottom left). Silencing was determined from cells co-expressing ectopic miR-19a/b (miR-19++, bottom right) and are reported in comparison to the MUT construct (set at 100%). (**B**) Cells were co-transfected as in (A) with a fixed amount (10 ng) of 3x-Bulge-miR-19 reporter and increasing amounts (0–200 ng) of Sponge-19-encoding construct. Cells were harvested and quantitative qRT-PCR analysis was performed on F-Luc reporter and Sponge-19 RNAs. Standard curves (indents) were generated using samples with spiked-in *in vitro* transcribed F-Luc and Sponge-19 RNAs of known concentrations. 3x-Bulge-miR-19 silencing (bottom panel) for each Sponge-19 concentrations was determined using luciferase assays as in (A). All data are presented as mean ± standard deviation from technical triplicates of three independent biological experiments. Two-tailed Student's *t*-test was used to calculate *P*-values (*: *P* < 0.05, **: *P* < 0.005).

Comparative dose-response data indicate that the stoichiometric ratio of endogenous miRISC pools to their reporters varies between miRNA families. While some are in excess and even saturate reporter silencing (miR-20 and miR-92), miR-26 miRISC populations are clearly sub-stoichiometric and cannot silence the reporter through slicing-independent mechanisms at endogenous concentrations. A subset represented by let-7 and miR-19 appears to program sufficient miRISC to lie in a dynamic range of functional concentrations. Silencing mediated by such miRNA concentrations should be responsive to mild concentration variations of both available miRISC and competing mRNA target sites. To test this hypothesis, we co-expressed ectopic miR-19a/b (Figure 5A, miR-19++) or an mRNA encoding miR-19 binding sites previously validated to function as a competing target RNA or sponge (Sponge-19) (22,23). We monitored and quantified the resulting miRISC availability (using 2′-O-Me capture) and reporter silencing (Figure 5A). Ectopic expression of miR-19a/b improved AGO2 miRISC capture by 3.3-fold, whereas co-expression of the cognate sponge at the highest concentration transfected resulted in 0.6-fold capture of miR-19 miRISC of control levels (Figure 5A, bottom right panel). Ectopic expression of additional miR-19 indeed resulted in 20% more silencing (Figure 5A, bottom right panel).

To determine the effect of impinged miRISC availability near reporter stoichiometry, we co-transfected increasing amounts of Sponge-19 (0.1–200 ng) with a fixed amount of 3x-Bulge-miR-19 reporter (10 ng). We precisely quantified the reporter mRNA and Sponge-19 RNA species under each condition (Figure 5B, upper panel) and the resulting repression (or de-repression) was measured (Figure 5B, lower panel). At its lowest concentration, Sponge-19 did not have any significant effect on the miR-19 reporter repression by miR-19, whereas at the highest concentration tested, Sponge-19 de-repressed 3x-Bulge-miR-19 by 40% (Figure 5B). At 10 ng of transfected Sponge-19 DNA, the resulting RNA reached near-stoichiometry with 3x-Bulge-miR-19 mRNA and drove a significant 20% de-repression of the reporter.

Altogether, these results experimentally demonstrate that under specific conditions of availability and stoichiometry with the co-expressed miRNA target sites, minor changes in miRISC availability can result in changes in silencing output.

## DISCUSSION

The main conclusions of this work are in line with a few previous studies in challenging the up-front assumption of linkage between miRNA expression and silencing activity (20,21,36,38). Using a unique design of miRISC capture and experimental alterations in miRISC availability, we add empirical evidence to a model wherein specific conditions of abundance, availability and stoichiometry with target sites have to be met for changes in miRNA expression or target site competition to affect mRNA silencing. Our data also support the emerging view that such requirements are only fulfilled for a subset of miRNAs (20,21). The consequences of these strengthened conclusions are important, as functional validation of predicted miRNA targets is still largely conducted under ectopic and over-expression conditions. As such, several of the biological functions that had been allotted to changes in miRNA expression or competitive effects between miRNA-binding sites will have to be revisited.

Previous work, and the results of this manuscript identify four reasons for disconnect between miRNA expression and their silencing activity; fragmentation in biochemically distinct complexes, sub-cellular localization of miRISC, target site competitive effects and direct stoichiometric titration of miRNAs by mRNAs. First, fragmentation of the miRISC pool is suggested by the disagreement between miRISC capture and the expression of miRNA families (Figure 2). It is further implicated by the results of miRISC titration on the silencing output (Figure 4): IC_50_ values follow the overall trend of silencing, but do not always reflect it. Part of this fragmentation is likely the result of non-homogenous distribution of mature miRNAs among complexes, rendering a significant fraction unavailable for direct involvement in silencing and capture using pull-down methods. One possible mechanism for this is the existence of one or several miRISC-like complexes with distinct biochemical behaviour. This possibility was recently supported in *Drosophila melanogaster* cells and in primary mammalian tissue (39,40), and may also occur in the course of an alternative mechanism of miRISC assembly (41). We note that since our experiments were limited to HEK 293T cells, it is possible that the composition of miRNA-associated complexes, and thus miRNA functional availability, may differ substantially in other systems. A second mechanism is fragmentation of the miRISC pool by sub-cellular localization. At least a sub-set of miRNAs, which includes miR-16, localizes to the nucleus and the importance of this fraction varies largely across miRNA families (20). P-bodies, wherein at least part of miRISC components localize (42,43), are another candidate structure for the sequestration of miRNAs. However, to this day it is still unclear if- or to what extent P-bodies partition the target recognition and silencing functions of the miRISC.

Results from our miRISC capture and dose-response assays highlight the potential importance of target site competition in modulating the output of miRNAs, but also point to limiting conditions of miRISC availability, and target site stoichiometry. Notwithstanding those important parameters, a recent study examined the contribution of target site affinity in potentiating target site competitive effects (21). Even though imperfect base-pairing miRNA-binding sites allow a single miRNA to regulate multiple target mRNAs (38), kinetics of miRISC-target site association suggest that exchange rate is slow in comparison to perfectly base-pairing sites, which dictate a slicing-dependent mechanism (44). Sequence and positioning of the base-pairing nucleotides of miRNAs with natural miRNA-binding sites can modulate the affinity of miRISC and hence modulate exchange rates (45). As miRNAs co-evolved with cognate target site sequences, the kinetics of exchange between competing target sites may be attuned to regulate the silencing potential on the critical mRNAs involved. Hastened exchange of miRISC between target sites could limit the functional competition between target sites, whereas high affinity association to a specific target site sequence/structure could potentiate target site competition effects. It is tempting to imagine that miRNA-binding sites encoded in naturally occurring competing RNA species, such as circular RNAs and pseudogenes, co-evolved with miRNAs (whether an entire family or a specific miRNA) to more efficiently trap and retain bound miRISC. As our experimental designs were largely based on 2′-O-Me oligonucleotides, which may not strictly reflect affinities of native miRNA-binding sites, we did not systematically examine the importance of target site affinity here. As such, it will be informative to extend quantitation of non-slicing exchange rates for each miRNA families in a complex competitive context, in a defined cellular transcriptome.

The titration curves presented in Figure 4 are strikingly reminiscent of the threshold model of miRNA molecular titration proposed by Mukherji *et* *al*. (12) and provide another explanation for the discordance between miRNA expression and their output. Modest changes to a defined miRISC pool, while prevailing at a lower concentration than its cognate target mRNA, would be expected to have limited effect on target silencing. Conversely, an mRNA bearing target sites saturated with excess miRISC should be buffered from changes in expression and/or availability. In this line of thought, the very fact that miRNA-mediated silencing is saturable by miRISC bears important mechanistic and biological implications. Saturation clearly raises the aspect of redundancy for distinct miRISC pools, programmed by distinct miRNA families, when they co-occupy the same target mRNA. Even if the availability of a particular miRISC pool is in the dynamic concentration range with an mRNA target, saturation of a second or even more target sites should pre-empt sensitive changes in silencing outcome.

We postulate that the different scenarios of expression, availability and stoichiometry experimentally revealed here can be selected to serve distinct physiological purposes and may unlock different properties of the miRISC machinery. Extreme abundance of a simple miRISC pool, programmed by a single miRNA family, such as miR-430 in the zebrafish embryo (46), is a logical fit for the rapid clearance of maternal transcripts at Maternal-to-Zygotic Transition (MZT). A different scenario should prevail in fully differentiated somatic cells reaching a homeostatic state. In this case, modest to moderate changes in expression of miRNAs, several of which may act redundantly, will rarely result in a drastic phenotype. Nonetheless, a subset of miRNAs should lie in the dynamic or responsive range of concentrations, with the available pool of miRISC being near-stoichiometric with biologically critical mRNA targets to allow a sensitive modulation of silencing in response to environmental and signalling cues. Re-visiting the properties of the miRISC in each of these states will potentially resolve yet more complexity in miRNA-mediated silencing mechanisms.

## Supplementary Material

SUPPLEMENTARY DATA
