# Supplementary material for: On the availability of microRNA-induced silencing complexes, saturation of microRNA-binding sites and stoichiometry
Source: Nucleic Acids Res. 2015 Jul 30;43(15):7556–65. doi: 10.1093/nar/gkv720 (PMC4551941; doi:10.1093/nar/gkv720)
Supplement: SUPPLEMENTARY DATA [file supp_43_15_7556__index.html]

On the availability of microRNA-induced silencing complexes, saturation of microRNA-binding sites and stoichiometry — On the availability of microRNA-induced silencing complexes, saturation of microRNA-binding sites and stoichiometry — SUPPLEMENTARY DATA 

# On the availability of microRNA-induced silencing complexes, saturation of microRNA-binding sites and stoichiometry

## SUPPLEMENTARY DATA

- SUPPLEMENTARY DATA
