# Supplementary material for: On the availability of microRNA-induced silencing complexes, saturation of microRNA-binding sites and stoichiometry
Source: Nucleic Acids Res. 2015 Jul 30;43(15):7556–65. doi: 10.1093/nar/gkv720 (PMC4551941; doi:10.1093/nar/gkv720)

Supplementary information

On the availability of microRNA-induced silencing complexes, saturation of microRNA-binding sites, and stoichiometry

Vinay K. Mayya and Thomas F. Duchaine

Supplementary figure legends

Supplementary figure S1. 2′-*O*-Me miRISC capture in HEK 293T cells. miRISC capture was conducted as explained in Figure 1A for the miR-19, -20, -92, -26 and let-7 families. Captured AGO1-4 **(A)** or AGO1 **(B)** were quantified and normalized relatively to miR-19 capture for each individual lysate (Bottom) from two independent biological replicates.

Supplementary figure S2. (A) Alignment of all known family members of miRNAs considered in this study. The seed region is highlighted in grey. Bases that differ from consensus are depicted in lowercase. **(B)** Quantification of northern analyses shown in Figure 2B. Results are presented as percentages of miRNA present in unbound fractions following RISC capture. These are compared to Input which is set to 100% for comparison. All data are presented as mean ± standard deviation of two biological independent experiments.

Supplementary figure S3. Quantitative analyses on the HEK 293T miRNAs examined in this study. **(A)** Standard curves were generated using RNA oligonucleotides, and used to quantify miR-19b, miR-20a, miR-92a and let-7a. **(B)** Northern blot quantification of miR-17, miR-20b, miR-25, miR-93, miR-106b and

let-7b for three independent biological replicates. **(C)** Hybridisation probe specificity is assessed by northern blot analysis of miR-20a in MEFs deficient of miR-17~92 cluster. 28S and 18S rRNA were used as loading controls. **(D)** qRT-PCR analyses of each miRNA for three independent biological replicates. RNA oligonucleotides were used as standards and copy numbers were calculated per microgram of isolated RNA. **(E)** Relative miRNA expression levels between northern blot analyses and qRT-PCR analyses. Values are normalised against miR-92a copy numbers.

Supplementary figure S4. Luciferase reporter silencing and de-repression assays on 3x-Bulge reporters. (Left panel) HEK 293T cells were co-transfected with 3x-Bulge reporters and increasing concentrations (0-66nM) of 2′-*O*-Me inhibitors for the indicated miRNAs. FLuc-3x-Bulge/R-Luc ratios are compared to F-Luc only (no binding site)/R-Luc, set as 100%. (Right panel) 3x-Bulge reporters were co-transfected with increasing concentrations (0-3.3nM) of dsRNA miRNA mimics for the indicated miRNAs. All data are presented as average ± standard deviation from technical triplicates of at-least three independent biological experiments.

Supplementary Table S1. List of oligonucleotides used in this study

F-Luc-T7-Fwd	GAAATTAATACGACTCACTATAGGGAATCCGGTACTGTTGGT
pmiRGLO-Rev	TGCCGGTCAGTCCTTTAGGC
GFP-T7-Fwd	GAAATTAATACGACTCACTATAGGGTTTAGTGAACCGTCA
pTRIPZ-Rev	GATGTGGAATGTGTGCGAGG
F-Luc-qPCR-Fwd	CGAGCACTTCTTCATCGTGG
F-Luc-qPCR-Rev	GAAGATGTTGGGGTGTTGCA
GFP-qPCR-Fwd	GTGACCACCCTGACCTACG
GFP-qPCR-Rev	TTGAAGAAGATGGTGCGCTC
Gene Specific_17/20a	CATGATCAGCTGGGCCAAGACTACCTGCAC
Gene Specific_19b	CATGATCAGCTGGGCCAAGATCAGTTTTGC
Gene Specific_92a	CATGATCAGCTGGGCCAAGAACAGGCCGGG
Gene Specific_106b	CATGATCAGCTGGGCCAAGAATCTGCACTG
Gene Specific_25	CATGATCAGCTGGGCCAAGATCAGACCGAG
Gene Specific_let7a	CATGATCAGCTGGGCCAAGAAACTATACAA
Gene Specific_let7b	CATGATCAGCTGGGCCAAGAAACCACACAA
Universal qPCR	CATGATCAGCTGGGCCAAGA
LNA_miR-17	C+AA+AGTGCTTACAGT
LNA_miR-20a	T+AA+AGTGCTTATAGT
LNA_miR-19b	T+GT+GCAAATCCATGC
LNA_miR-92a	T+AT+TGCACTTGTCCC
LNA_miR-106b	T+AA+AG+TGCTGACAG
LNA_miR-25	C+AT+TGCACTTGTCTC
LNA_miR-let7a/b	T+GA+GGTAGTAGGTTG
1xPer-miR19b Fwd	CTAGATCAGTTTTGCATGGATTTGCACAGC
1xPer-miR19b Rev	GGCCGCTGTGCAAATCCATGCAAAACTGAT
1xPer-mut miR19b Fwd	CTAGATCAGTTTTGCAACCATTTCGTCAGC
1xPer-mut miR19b Rev	GGCCGCTGACGAAATGGTTGCAAAACTGAT
3x-Bulge-miR19 Fwd	CTAGATCAGTTTTGCAATTTGCACACCGCGGTATCAGTTTTGCAATT TGCACAGAATTCATATGTCAGTTTTGCAATTTGCACAGC
3x-Bulge-miR19 Rev	GGCCGCTGTGCAAATTGCAAAACTGACATATGAATTCTGTGCAAAT TGCAAAACTGATACCGCGGTGTGCAAATTGCAAAACTGAT
3x-Bulge-mutmiR19 Fwd	CTAGATCAGTTTTGCAATTTCGTCACCGCGGTATCAGTTTTGCAATT TCGTCAGAATTCATATGTCAGTTTTGCAATTTCGTCAGC
3x-Bulge-mut miR19 Rev	GGCCGCTGACGAAATTGCAAAACTGACATATGAATTCTGACGAAAT TGCAAAACTGATACCGCGGTGACGAAATTGCAAAACTGAT
1xPer-miR-20a Fwd	CTAGACTACCTGCACTATAAGCACTTTAGC
1xPer-miR-20a Rev	GGCCGCTAAAGTGCTTATAGTGCAGGTAGT
1xPer-mut miR-20a Fwd	CTAGACTACCTGCACTTATAGCAGAATAGC
1xPer-mut miR-20a Rev	GGCCGCTATTCTGCTATAAGTGCAGGTAGT
3x-Bulge-miR20 Fwd	CTAGACTACCTGCACTAGCACTTTACCGCGGTACTACCTGCACTAGC ACTTTAGAATTCATATGCTACCTGCACTAGCACTTTAGC
3x-Bulge-miR20 Rev	GGCCGCTAAAGTGCTAGTGCAGGTAGCATATGAATTCTAAAGTGCT AGTGCAGGTAGTACCGCGGTAAAGTGCTAGTGCAGGTAGT
3x-Bulge-mutmiR20 Fwd	CTAGACTACCTGCACTAGCAGAATACCGCGGTACTACCTGCACTAG CAGAATAGAATTCATATGCTACCTGCACTAGCAGAATAGC

3x-Bulge-mut miR20 Rev	GGCCGCTATTCTGCTAGTGCAGGTAGCATATGAATTCTATTCTGCTAGTGCAGGTAGTACCGCGGTATTCTGCTAGTGCAGGTAGT
1xPer-let-7a Fwd	CTAGAAACTATACAACCTACTACCTCAGC
1xPer-let-7a Rev	GGCCGCTGAGGTAGTAGGTTGTATAGTTT
1xPer-mut let-7a Fwd	CTAGAAACTATACAAGGAACTAGGACAGC
1xPer-mut let-7a Rev	GGCCGCTGTCCTAGTTCCTTGTATAGTTT
3x-Bulge-let-7a Fwd	CTAGAAACTATACAAACTACCTCACCGCGGTAAACTATACAAACTACCTCAGAATTCATATGAACTATACAAACTACCTCAGC
3x-Bulge-let-7a Rev	GGCCGCTGAGGTAGTTTGTATAGTTCATATGAATTCTGAGGTAGTTTGTATAGTTTACCGCGGTGAGGTAGTTTGTATAGTTT
3x-Bulge-mut let-7a Fwd	CTAGAAACTATACAAACTAGGACACCGCGGTAAACTATACAAACTAGGACAGAATTCATATGAACTATACAAACTAGGACAGC
3x-Bulge-mut-let-7a Rev	GGCCGCTGTCCTAGTTTGTATAGTTCATATGAATTCTGTCCTAGTTTGTATAGTTTACCGCGGTGTCCTAGTTTGTATAGTTT
1xPer-let-7b Fwd	CTAGAAACCACACAACCTACTACCTCAGC
1xPer-let-7b Rev	GGCCGCTGAGGTAGTAGGTTGTGTGGTTT
1xPer-mut let-7b Fwd	CTAGAAACCACACAAGGAACTAGGACAGC
1xPer-mut let-7b Rev	GGCCGCTGTCCTAGTTCCTTGTGTGGTTT
3x-Bulge-let-7b Fwd	CTAGAAACCACACAAACTACCTCACCGCGGTAAACCACACAAACTACCTCAGAATTCATATGAACCACACAAACTACCTCAGC
3x-Bulge-let-7b Rev	GGCCGCTGAGGTAGTTTGTGTGGTTCATATGAATTCTGAGGTAGTTTGTGTGGTTTACCGCGGTGAGGTAGTTTGTGTGGTTT
3x-Bulge-mut let-7b Fwd	CTAGAAACCACACAAACTAGGACACCGCGGTAAACCACACAAACTAGGACAGAATTCATATGAACCACACAAACTAGGACAGC
3x-Bulge-mut let-7b Rev	GGCCGCTGTCCTAGTTTGTGTGGTTCATATGAATTCTGTCCTAGTTTGTGTGGTTTACCGCGGTGTCCTAGTTTGTGTGGTTT
1xPer-miR-26a Fwd	CTAGAAGCCTATCCTGGATTACTTGAAGC
1xPer-miR-26a Rev	GGCCGCTTCAAGTAATCCAGGATAGGCTT
1xPer mut miR-26a Fwd	CTAGAAGCCTATCCTCCTTTACAACAAGC
1xPer-mut miR-26a Rev	GGCCGCTTGTTGTAAAGGAGGATAGGCTT
3x-Bulge-miR-26Fwd	TCGAGCCTATCCTTTACTTGAAGAATTCCCTATCCTTTACTTGAAGAATTCCCTATCCTTTACTTGAAGC
3x-Bulge-miR-26 Rev	GGCCGCTTCAAGTAAAGGATAGGGAATTCTTCAAGTAAAGGATAGGGAATTCTTCAAGTAAAGGATAGGC
3x-Bulge-mut miR-26 Fwd	TCGAGCCTATCCTTTACAACAAGAATTCCCTATCCTTTACAACAAGAATTCCCTATCCTTTACAACAAGC
3x-Bulge-mut miR-26 Rev	GGCCGCTTGTTGTAAAGGATAGGGAATTCTTGTTGTAAAGGATAGGGAATTCTTGTTGTAAAGGATAGGC

Mayya & Duchaine, Supplementary Figure S1

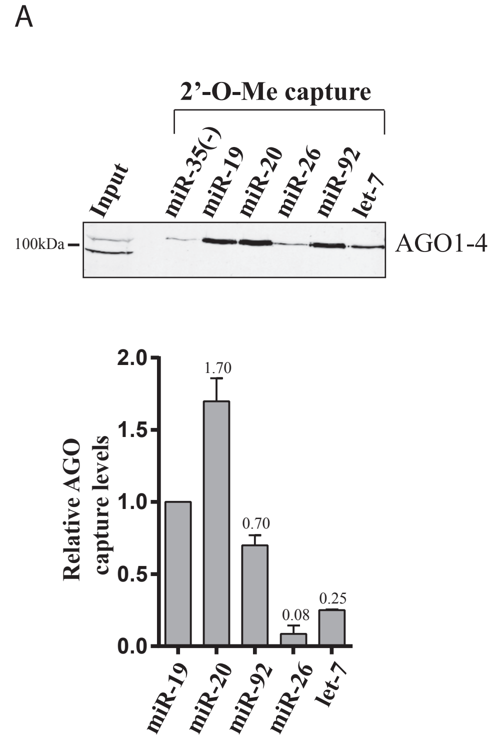

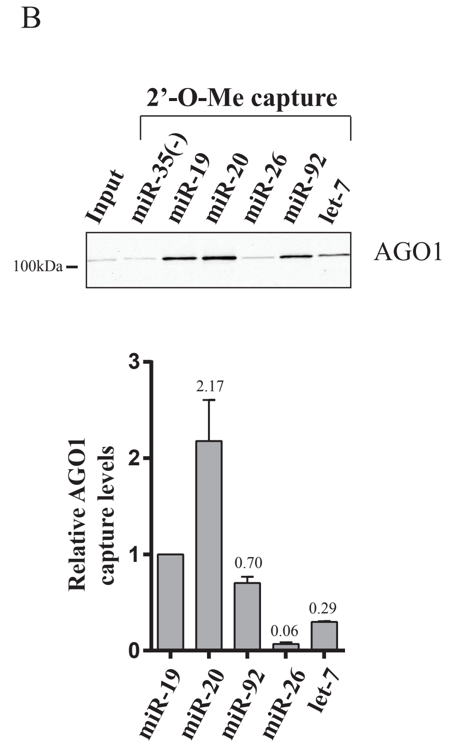

Mayya & Duchaine, Supplementary Figure S2

A

hsa-miR-19b	UGUGCAAAUCCAUGCAAAACUGA
hsa-miR-19a	UGUGCAAAUCuAUGCAAAACUGA
hsa-miR-20a	UAAAGUGCUUAUAGUGCAGGUAG
hsa-miR-20b	cAAAGUGCUcAUAGUGCAGGUAG
hsa-miR-17	cAAAGUGCUUAcAGUGCAGGUAG
hsa-miR-106a	aAAAGUGCUUAcAGUGCAGGUAG
hsa-miR-106b	UAAAGUGCUgAcAGUGCAGaU
hsa-miR-93	cAAAGUGCUguUcGUGCAGGUAG
hsa-miR-92a	UAUUGCACUUGUCCCGGCCUGU
hsa-miR-92b	UAUUGCACUcGUCCCGGCCUcc
hsa-miR-25	cAUUGCACUUGUCuCGGuCUGa
hsa-miR-32	UAUUGCACaUuaCuaaGuugca
hsa-miR-363	UAUUGCACUUGUCCCGGCCUGU
hsa-miR-367	UAUUGCACUUGUCCCGGCCUGU
hsa-let-7a	UGAGGUAGUAGGUUGUAUAGUU
hsa-let-7b	UGAGGUAGUAGGUUGUgUgGUU
hsa-let-7c	UGAGGUAGUAGGUUGUAUgGUU
hsa-let-7d	aGAGGUAGUAGGUUGcAUaGUU
hsa-let-7e	UGAGGUAGgAGGUUGUAUAGUU
hsa-let-7f	UGAGGUAGUAGaUUGUAUAGUU
hsa-let-7g	UGAGGUAGUAGuUUGUAcAGUU
hsa-let-7i	UGAGGUAGUAGuUUGUgcuGUU

B

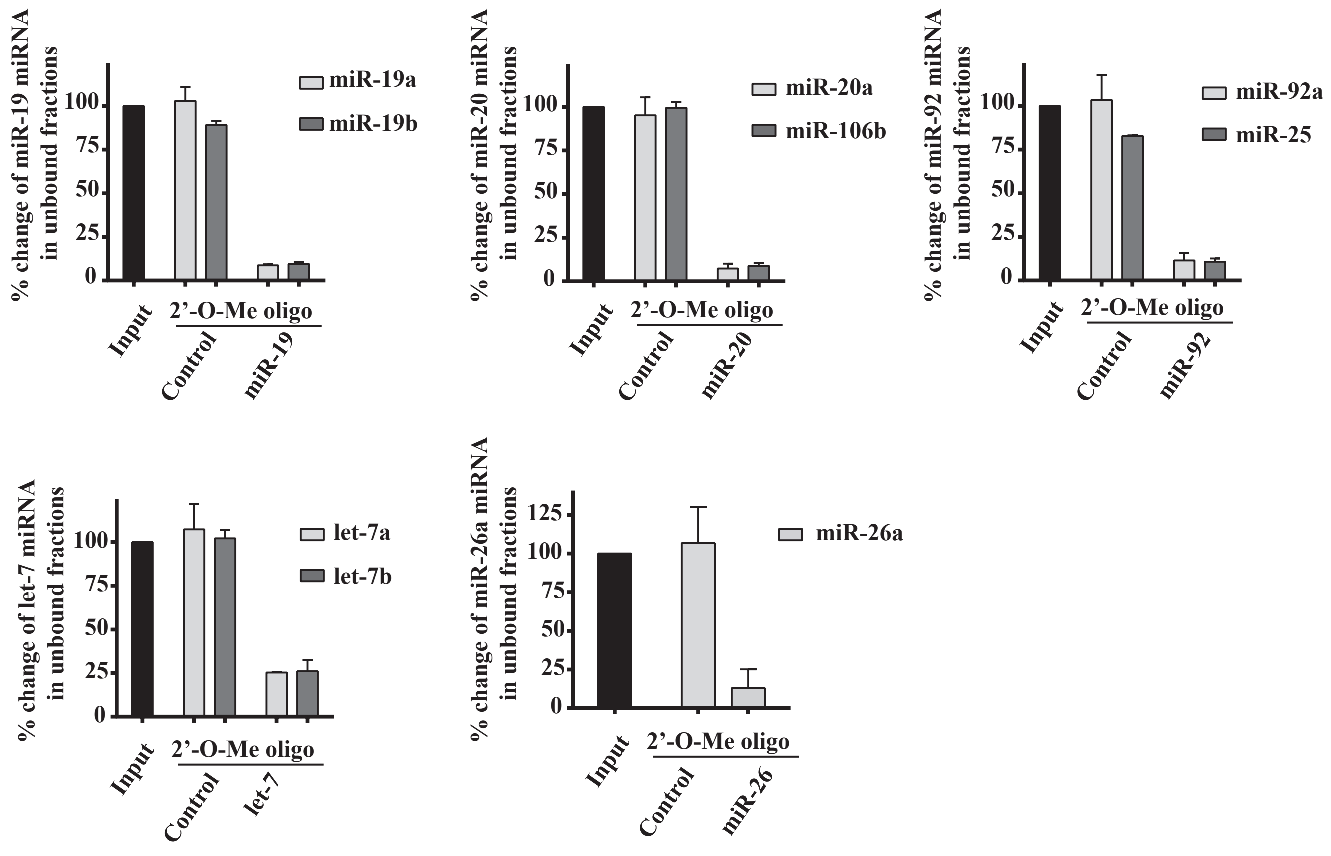

Mayya & Duchaine, Supplementary Figure S3

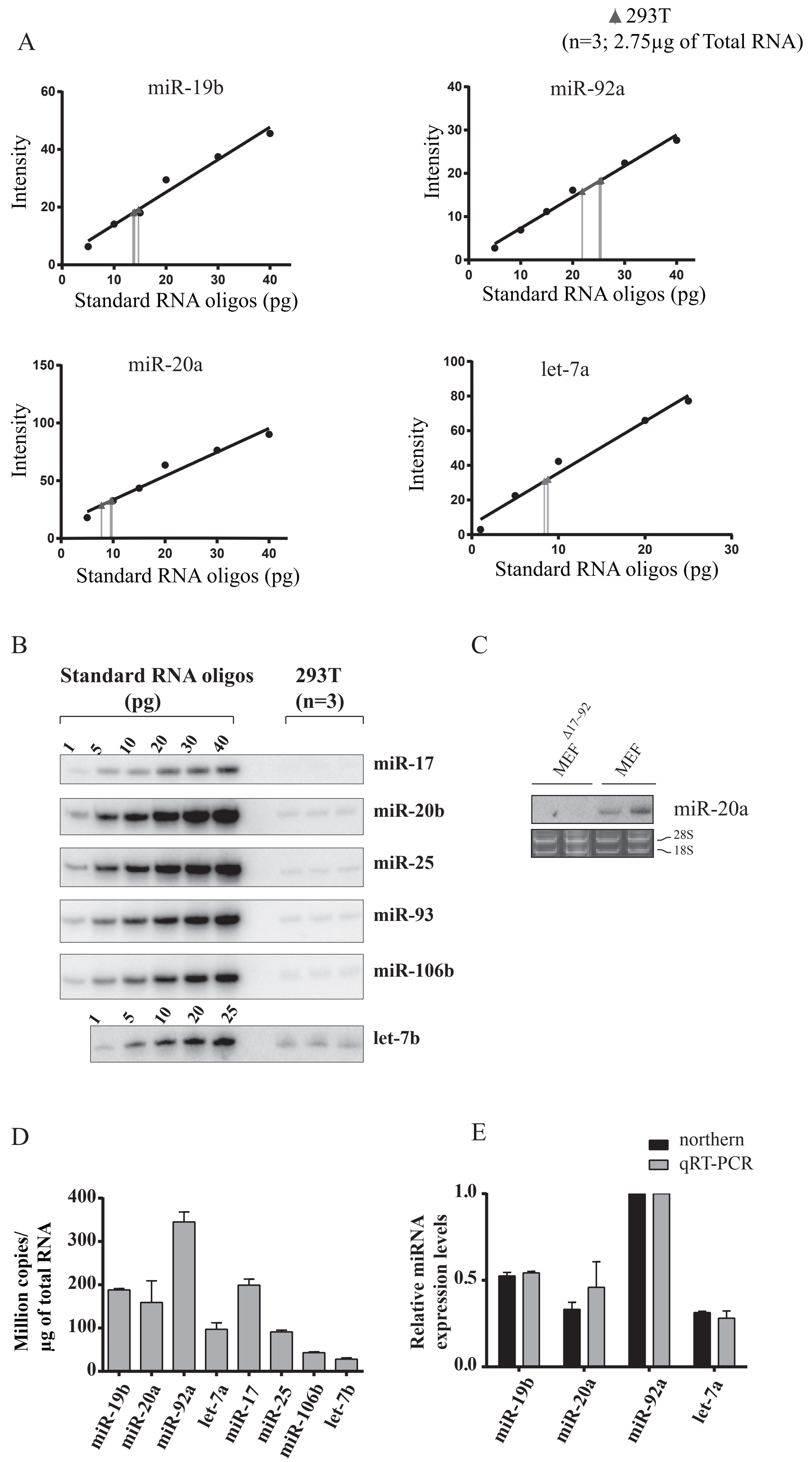

Mayya & Duchaine, Supplementary Figure 4

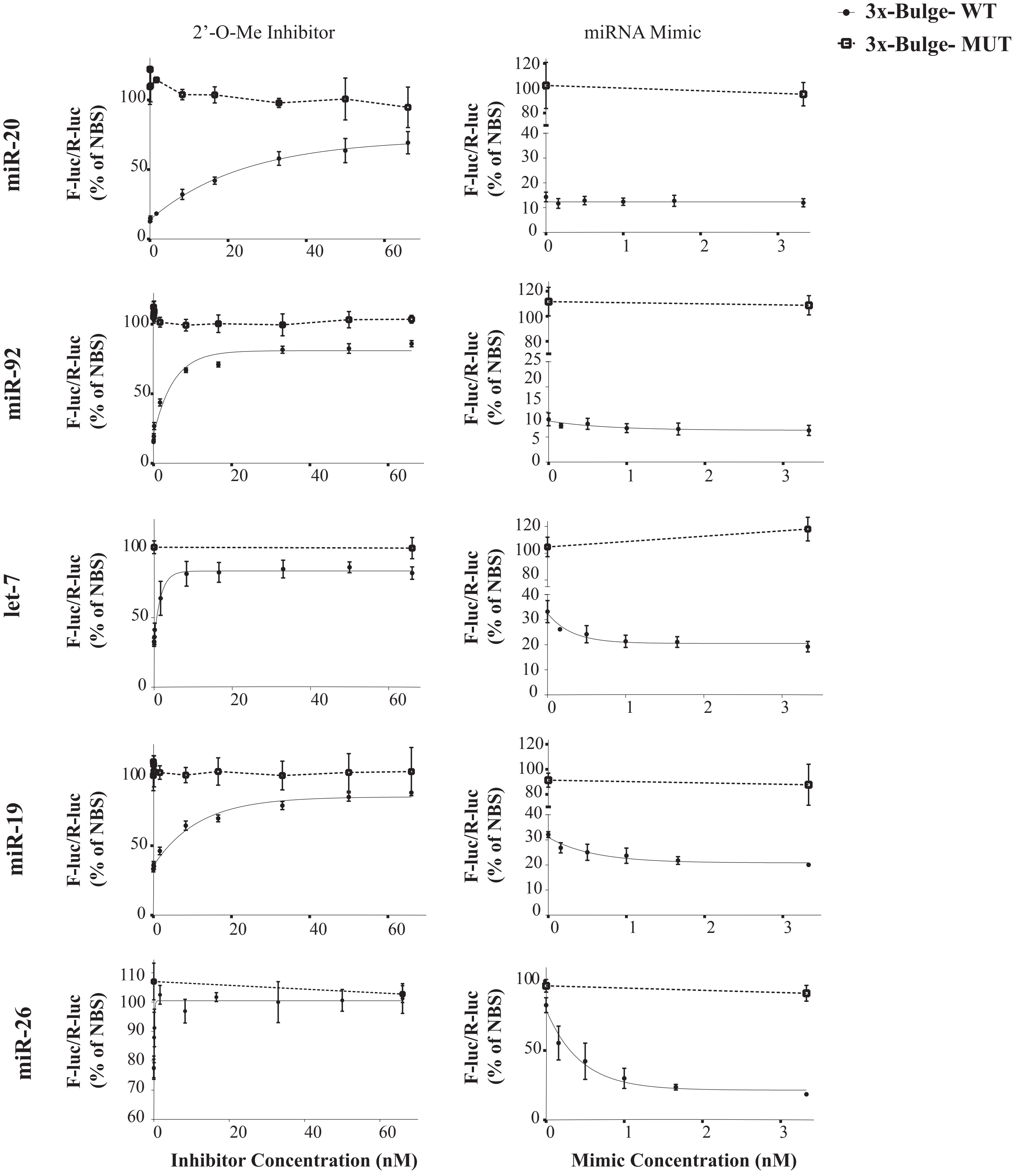

Supplement: SUPPLEMENTARY DATA [file supp_gkv720_nar-00556-a-2015-File007.pdf]
